# High performance polymer tandem solar cell

**DOI:** 10.1038/srep18090

**Published:** 2015-12-16

**Authors:** Wilson Jose da Silva, Fabio Kurt Schneider, Abd. Rashid bin Mohd Yusoff, Jin Jang

**Affiliations:** 1 Department of Information, Display and Advanced Display Research Center, Kyung Hee University, Dongdaemun-ku, Seoul, 130-171 Republic of Korea; 2 Universidade Tecnologica Federal do Parana, GPGEI – Av. Sete de Setembro, 3165 – CEP 80230-901 – Curitiba, Parana, Brasil

## Abstract

A power conversion efficiency of 9.02% is obtained for a fully solution-processed polymer tandem solar cell, based on the diketopyrrolopyrrole unit polymer as a low bandgap photoactive material in the rear subcell, in conjunction with a new robust interconnecting layer. This interconnecting layer is optically transparent, electrically conductive, and physically strong, thus, the charges can be collected and recombined in the interconnecting layer under illumination, while the charge is generated and extracted under dark conditions. This indicates that careful interface engineering of the charge-carrier transport layer is a useful approach to further improve the performance of polymer tandem solar cells.

Back in 2011, Mitsubishi Chemical established a 10% efficiency of single junction organic photovoltaic (OPV)^1^ Later, Heliatek and Yang and their respective co-workers also announced double digits efficiency for their multiple junctions OPVs^2^,^3^,^4^. Since then, we have seen fast progress in OPV, especially in terms of anode or cathode interfacial layers, active layers (ternary systems), and in synthesizing low bandgap polymers. The former usually offered some advantages including high short-circuit current density (J_SC_), as well as complementary absorption of up to 1000 nm. However, it is rather difficult to obtain an excellent low bandgap polymer which can offer both high J_SC_ and also high V_OC_ of > 0.8 V, while providing complementary absorption. Until recently, Mitsubishi Chemical has had great success in single junction PV, where they announced an NREL certified efficiency of 11.1%^5^. Such a large jump from 10 to 11.1% must be from an exceptionally high quality polymer or small molecule. In addition, several groups have demonstrated a triple-junction polymer and small molecule PVs exceeding 11% PCE^6^,^7^,^8^. Triple-junction PVs in particular have been proposed to make full use of the 1 + 1 + 1 device configuration employing wide, medium, and low bandgap materials as the front, middle, and bottom subcells, respectively^6^. However, an excellent donor material is required in order to achieve high performance triple-junction PV. In this research field, chemists usually employ either benzothiadiazole (BT)^9^, diketopyrrolopyrrole (DPP)^10^,^11^,^12^, isodingo^13^, or terthiophene^14^, units as the pillar in designing high quality low bandgap polymers.

Recently, Y. Yang and R. A. J. Janssen, with their respective co-workers, were able to produce excellent and good low bandgap polymers, where Yang *et al.* introduced two strong electron-withdrawing fluorene atoms onto the benzothiadiazole (BT) unit, to form the difluorobenzothiadiazole (DFBT) unit in order to lower the highest occupid molecular orbital (HOMO)^9^. On the other hand, Janssen *et al.* developed a low bandgap polymer with diketopyrrolopyrrole (DPP) as an electron-poor conjugated unit, alternating with a terthiophene unit, which is electron-rich and can absorb well into the NIR region (960 nm)^10^

Hence, at this point, it is clear that both the low bandgap polymer and multiple junction OPVs concepts have proven viable. These will most likely be found in future incarnations of OPVs, provided that the appropriate metal electrode can be coated by printing technology. In the present study, we describe a method for preparing inverted tandem solar cells which are compatible with an all printable process. In general, there are a few issues in achieving high performance polymer tandem solar cells which go beyond the processing and optimizing of a single junction OPV^11^,^12^ Primarily, the fabrication of polymer tandem solar cells usually consists of two and sometimes more active materials. It is challenging since these active materials usually are prepared from comparable organic solvents. Thus, to prevent the underneath front subcell from dissolving again during the deposition of a rear subcell, the middle electrode, or the so-called interconnecting layer (ICL), must be processed from an orthogonal solvent, typically some aqueous solution or suspension. Solution processed electron transport materials, namely zinc oxide (ZnO) and titanium dioxide (TiO_2_) have been introduced as n-type buffer layer in organic photovoltaic devices^10^,^11^,^12^,^15^,^16^. Meanwhile, a p-type material poly(3,4-ethylenedioxythiophene) polystyrene sulfonate (PEDOT:PSS) has been widely used to transport the hole from active layer to its respective electrode. Nonetheless, our commonly used PEDOT:PSS is not able to give perfect protection. Thus, Yang *et al.* introduced surfactant and dimethylformamide (DMF) into PEDOT:PSS in order to improve its mechanical properties and conductivity, which lead to good transparency and conductivity^14^,^17^. In their study, a PCE of 5.84% has been reported. Two years later, Dou *et al.* demonstrated and significant improvement, where the PCE improved to 8.62% utilizing a modified PEDOT:PSS/ZnO ICL^14^. In short, a perfect ICL is needed to avoid any potential drop, otherwise the V_OC_ of the tandem device will be affected. Here, we introduced a new concept ICL, combining our commonly used p-type hole transport material PEDOT:PSS mixed graphene oxide (GO) paired with an n-type material lithium zinc oxide (LZO). The fabricated device employing this ICL illustrates a high V_OC_ of about 1.60 V.

## Results and Discussion

**Figure 1:**
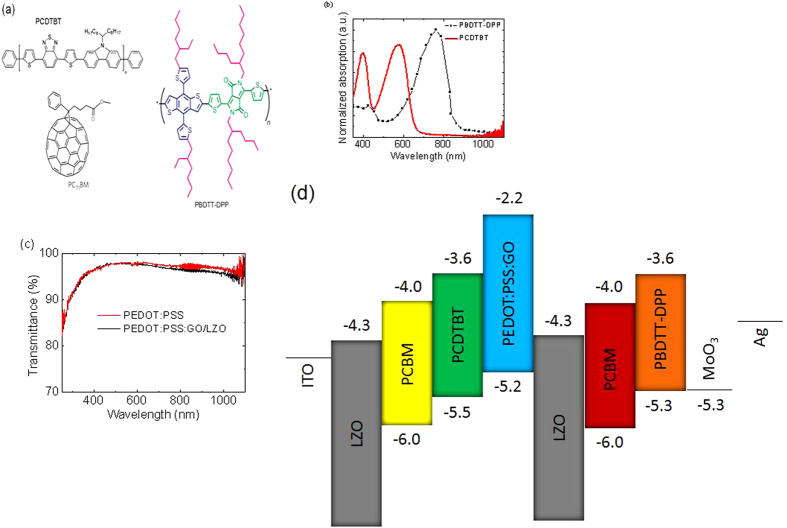
(**a**) Chemical structure of the high bandgap (PCDTBT), low bandgap (PBDTT-DPP) polymers and fullerene used in inverted tandem solar cells. (**b**) UV-Visible absorption spectra of PCDTBT and PBDTT-DPP films. (**c**) Transmittance spectra of PEDOT:PSS and PEDOT:PSS:GO/LZO. (**d**) Energy band diagram of the inverted tandem cell.

Here we demonstrate solution processable polymer tandem solar cells consisting of two different active materials. Our polymer tandem solar cells, which consist of two subcells along with complimentary absorption spectra^18^,^19^, allow us to harvest as many photons as possible (Fig. 1a). These two subcells are physically separated by PEDOT:PSS:GO/LZO as the ICL. The ICL functions based on various different reasons including (i) a charge recombination zone, and (ii) a shielding layer for the front subcell during the deposition of the rear subcell. In general, because these subcells have completely different absorption spectra, high-energy photons are absorbed in the front subcell, while low-energy photons are absorbed in the rear subcell; thus the whole visible region of the solar spectrum can be covered. The subcells of our tandem device are electronically connected in series, in which the V_OC_ of the polymer tandem solar cells is the summation of the V_OC_’s of the front and rear subcells. Attenuation of incident light on the rear subcell, caused by absorption and the optical cavity of the front subcell, leads to a lower photocurrent density in the rear subcell, which determines the photocurrent density of the polymer tandem solar cells. Therefore, we introduce in this study a commercially available DPP unit-based low bandgap (*E*_g_ ≈ 1.44 eV) polymer, poly{2,6′-4,8-di(5-ethylhexylthienyl)benzo[1,2-b;3,4-b]dithiophene-alt-5-dibutyloctyl-3,6-bis(5-bromothiophen-2-yl)pyrrolo[3,4-c]pyrrole-1,4-dione} (PBDTT-DPP)^10^,^11^,^12^ (Fig. 1b) incorporating a wide-bandgap (*E*_g_ ≈ 1.9 eV) polymer, poly(N-9′-heptadecanyl-2,7-carbazole-alt-5,5-(4′,7′-di-2-thienyl-2′,1′,3′-benzothiadiazole) (PCDTBT) (Fig. 1b)^20^, as the front cell, and PEDOT:PSS:GO/LZO layer as the interconnecting layer (ICL) into tandem solar cells; the devices achieve PCEs of 9.02%, with a *J*_SC_ of 8.53 mA/cm^2^, VOC of 1.60 V, and fill factor (FF) of 66.14%. The results show that PBDTT-DPP is an ideal low bandgap polymer to fabricate high efficiency solution-processed polymer tandem solar cells. Moreover, the results elucidate that the interfacial engineering of the charge-carrier recombination layer is a useful approach to further improving the PCEs of polymer tandem organic solar cells.

Figure 1b shows the absorption spectra of PCDTBT:PC_70_BM and PBDTT-DPP:PC_71_BM layers as active layers for the front and rear subcells. The absorption of the PCDTBT:PC_70_BM layer covers from 300 to 700 nm with strong absorption located at the range of 350–600 nm. The absorption of the PCDTBT:PC_70_BM layer covers the visible spectrum range from 400 to 650 nm, just falling in the valley of the PBDTT-DPP:PC_71_BM absorption spectrum. In order to ensure that more photons pass through the middle ICL and efficient light absorption in the rear subcell, the PBDTT-DPP:PC_71_BM film is employed as the active layer of the rear subcell. The complementary absorption of the PCDTBT:PC_70_BM and PBDTT-DPP:PC_71_BM layers in the polymer tandem solar cells may efficiently improve the solar light harvesting. Combining these active materials, the absorption spectrum of the tandem photovoltaic cell covers almost the entire visible spectrum as well as the near infrared region. Thus, we anticipate that the improved absorption spectrum results in a larger number of photogenerated excitons and causes a higher photocurrent. Transmittance of the ICL of the PEDOT:PSS:GO/LZO, which electrically connects the front and rear subcells, is also shown in Fig. 1c. The transmittance is higher than 95% and below 650 nm. When the wavelength is above 650 nm, the transmittance is slightly less than 95%. The device structure and the corresponding energy diagram are shown in Fig. 1d. Lithium doped zinc oxide (LZO) was used as the electron-transport material because their work function matches well with the acceptors and high electron mobility^21^. PEDOT:PSS:GO was used as the hole-transport material for PCDTBT, and MoO_3_ was used for PBDTT-DPP because of its good work function alignment with polymer and its high hole mobility. Ultraviolet photoelectron spectroscopy (UPS) was used to examine the work functions of the ICLs, including PEDOT:PSS:GO and LZO (Fig. 1d). The energy difference between the different layers was minimized by material selections to ensure good charge transport.

**Figure 2:**
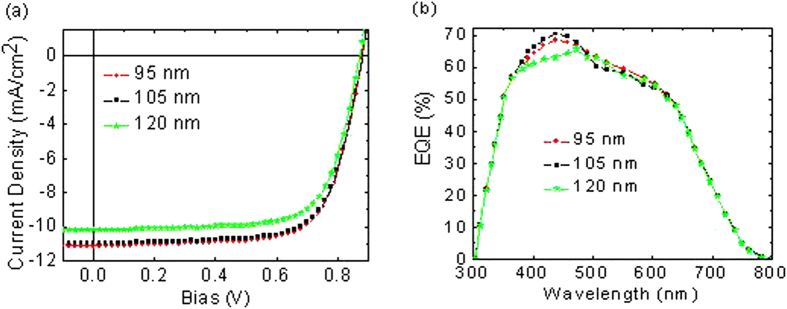
(**a**) J-V plots. (**b**) External quantum efficiency. The structure of the front subcell is ITO/LZO (30 nm)/PCDTBT:PC_70_BM (95 nm)/PEDOT:PSS (10 nm)/Ag (100 nm).

We carried out thickness optimization experiments of single junction OPVs based on PCDTBT:PC_70_BM (see Fig. 2a). The single junction OPV was fabricated on the ITO substrate of 0.2 cm × 0.2 cm dimension using a traditional sandwich structure of ITO/LZO (30 nm)/PCDTBT:PC_70_BM (95 nm)/PEDOT:PSS:GO (10 nm)/Ag (100 nm). The active layer thickness varied between 95 to 115 nm. All constructed single junction OPVs were characterized under standard spectral condition AM1.5 G at 100 mW/cm^2^. The PCDTBT:PC_70_BM thickness experiment shows major influence of the thickness on the J_SC_ and FF values (up to 11.13 mA/cm^2^ and up to 70.78%). The highest J_SC_ of 11.13 mA/cm^2^ was obtained from the device with a thickness of 95 nm. As shown in Table 1, the J_SC_ decreases as the active layer thickness increases. The J_SC,exp_ demonstrates a 8.53% decrease from 11.13 mA/cm^2^ (95 nm) to 10.18 mA/cm^2^ (120 nm), with increases in the active layer thickness. As we can see from Table 1, the J_SC,exp_ values are in good agreement with the J_SC,sim_ values obtained from numerical simulations, where the ratio of the J_SC,exp_ over J_SC,sim_ is virtually unity. Despite higher absorption in the thicker active layer, the J_SC_ significantly decreased to 10.18 mA/cm^2^ from 11.13 mA/cm^2^ with increased active layer thicknesses. This phenomenon can be attributed to at least two possibilities. First, the electric field inside the active materials slightly decreased as we increased the thickness from 95 to 120 nm. Hence, the dissociation rate of the excitons will decline at lower electric fields because this process relies upon the electric field (Table 1). Secondly, a thicker active layer also provides a long pathway for charge collection at their respective top and bottom electrodes. Consequently, one can also expect that there is a high probability for the separated charges to be recombined again before they arrive at their respective electrodes. The maximum J_SC_ clearly indicates that maximum optical interference is responsible for a field redistribution to increase absorption within the active layer^22^. The PCE decreases for the device with 120 nm due to a clearly reduced J_SC_ and reduced FF (see Table 1). The FF also decreased from 70.78% (95 nm) to 69.97% (120 nm) with an increased active layer thickness. The decrease in FF indicates that the probability of photogenerated charge carriers become trivial with thicker active material. It is also worth noting that at a short-current condition, where the maximum power point is smaller, there will be a lower dissociation rate as well as a higher recombination rate^23^. As mentioned above, in thicker active materials there will be longer pathways for the separated charges to be collected at the top and bottom electrodes. Thus, if the higher probabilities of these charges are recombined, it will result in a lower FF. The best single junction OPV based on PCDTBT:PC_70_BM depicts a PCE of 6.93% (see Table 1), which outperformed the previously published work of 6.2%^24^. Considering the rather narrow absorption band of the PCDTBT:PC_70_BM at short wavelengths (λ_max_ ≈ 450 nm, almost no absorption > 800 nm, see Fig. 1b). It is reported that the PCE of 6.93% is quite high and among the highest reported for the shorter wavelength range of solar irradiation^25^,^26^. Meanwhile, Fig. 2b shows the external quantum efficiency (EQE) spectra for the corresponding single junction OPVs, which demonstrate a wider response from 350 up to 850 nm, with an average EQE of 47%. The integrated J_SC_’s from the EQE spectra are within the 3% error; 11.02, 10.82, and 9.94 mA/cm^2^, respectively.

**Figure 3:**
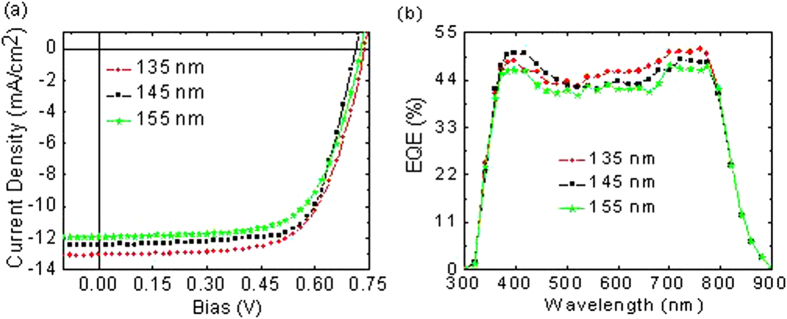
PBDTT-DPP:PC_71_BM-based single junction polymer solar cells characteristics with various active layer thicknesses. (**a**) J-V plots. (**b**) External quantum efficiency.

Figure 3a and Table 2 demonstrate the J-V characteristics of the rear subcell OPVs under 100 mW/cm^2^ at AM1.5 G illumination, with a device configuration ITO/LZO (30 nm)/PBDTT-DPP:PC_71_BM/MoO_3_ (15 nm)/Ag (100 nm) with PBDTT-DPP:PC_71_BM variation thicknesses of 135, 145, and 155 nm. In the PBDTT-DPP:PC_71_BM thickness study, V_OC_ remains constant at around 0.74 V for all thicknesses studied (Fig. 3b). Similar to that of PCDTBT:PC_70_BM devices, the J_SC,sim_ decreases with increased PBDTT-DPP:PC_71_BM thickness (see Table 2), as expected from the increased absorption. With 100 nm of PBDTT-DPP:PC_71_BM, the device performance enhance to FF = 65.69% and PCE = 6.36%. For an identical device, with a different ETL (ZnO:TiO_x_ = 30 nm), the device demonstrated a FF = 55.35% and a PCE of 2.52%. It is worth noting that for LZO ETL, both J_SC,exp_ and J_SC,sim_ are in agreement, leading to a ratio of ≈1. In addition, for thicker active layers (145 and 155 nm), the ratios decreased below 0.9. Thus, we conclude that as one increases the active layer thickness, the absorption increases but the J_SC_ continues to decrease, demonstrating that the increase in light absorption does not lead to a higher photocurrent under short-circuit conditions. This is accompanied with a plunge of FF slightly from 65.69 to 63.50%. This phenomenon occurred because of poor charge transport properties even though it is in relatively thick active layers. Above all, the thinnest device, of 100 nm, demonstrated the best performance with a high PCE of 6.36%, resulting from strong absorption of the PBDTT-DPP:PC_71_BM, even in thin films (see Fig. 1b).

Figure 3b depicts the EQE spectra of a PBDTT-DPP:PC_71_BM single junction OPVs with different active layer thicknesses. As we can see from the spectra, the J_SC_ values obtained from the integrated EQE are in agreement with the J_SC_ values obtained from the J-V characteristics (Fig. 3a). The values are 12.97, 12.37, and 11.88 mA/cm^2^ for 135, 145, and 155 nm, respectively.

These observations of PBDTT-DPP:PC_71_BM are a significant improvement compared to the previous study^14^, where they achieved a PCE of 6.12%, using PEDOT:PSS as the hole transport layer.

In the tandem geometry, the front and rear single junction organic solar cells are stacked in series, which implies that, for a well-performing tandem cell, the V_OC_ of the polymer tandem solar cell is equal to the sum of the V_OC_’s of both the front and rear subcells. The J_SC_ of the polymer tandem solar cells is limited by the lowest J_SC_ of the two individual subcells. For maximum performance of the polymer tandem solar cells, the J_SC_ of each subcell has to be matched. It is worth mentioning that, the front and rear subcells of the polymer tandem solar cells can also be measured individually by contacting the front (anode) and middle (cathode) electrodes for the front subcell, and the middle (anode) and top (cathode) electrodes for the rear subcell. As explained above, the combination of 95 nm PCDTBT:PC_70_BM for the front subcell (large bandgap) and 135 nm PBDTT-DPP:PC_71_BM for the rear subcell (small bandgap) results in an optimized optical and electronic coupling for the tandem cells in series. The 135 nm thickness of the active layer (PBDTT-DPP:PC_71_BM) of the rear subcell is optimized for performance. The structure of the polymer tandem solar cells is shown in Fig. 1d.

**Figure 4:**
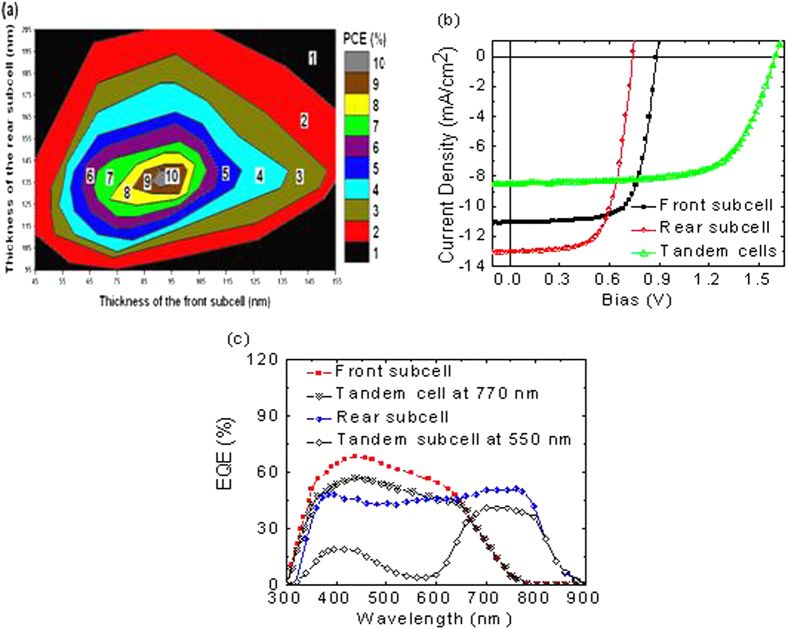
(**a**) Optical simulations results as a function of different thicknesses for the front and rear subcells. (**b**) J-V characteristics of the front, rear, and inverted tandem polymer solar cells. (**c**) EQE measured under relevant bias illumination conditions. The structure of our rear subcell is ITO/LZO (30 nm)/PBDTT-DPP:PC_71_BM/MoO_3_ (15 nm)/Ag (100 nm).

In order to guide the fabrication of the tandem solar cells, optical modeling using the transfer matrix formalism^27^ was performed on relevant device architectures. Figure 4a depicts the output of the simulated tandem device performance for the structure ITO/LZO (nm)/PCDTBT:PC_70_BM (nm)/PEDOT:PSS:GO (nm)/LZO (nm)/PBDTT-DPP:PC_71_BM (nm)/MoO_3_ (nm)/Ag (nm). The simulations assume a constant IQE and are meant to focus experimental efforts and are not an absolute predictor of device performance. Figure 4a shows that the simulations data as a function of different thicknesses for the front and rear subcells. One could achieve a maximum efficiency of 10% with suitable front and rear subcell thicknesses. Figure 4b presents the measured J-V characteristics of the front and rear subcells, along with the polymer tandem solar cells under 100 mW/cm^2^ AM1.5 illumination. The values of the V_OC_, J_SC_, and FF extracted from the measurements are summarized in Table 3. The front subcell single junction OPV demonstrates a V_OC_ of 0.88 V, J_SC_ of 11.13 mA/cm^2^, FF of 70.78%, and AM1.5 PCE of 6.93%. The rear subcell single junction OPV shows a V_OC_ of 0.74 V, J_SC_ of 13.08 mA/cm^2^, FF of 65.69%, and AM1.5 PCE of 6.36%.

The ideal polymer tandem solar cells should demonstrate a V_OC_ equal to the summation of the V_OC_’s of the front and rear subcells. The polymer tandem solar cells with PEDOT:PSS:GO/LZO ICL exhibit a V_OC_ of 1.60 V, which is 0.02 V less than the ideal summation of the V_OC_’s of the front and rear single junction OPVs. The polymer tandem solar cells provide an AM1.5 PCE of 9.02%, compared to the 6.93 and 6.36% values achieved by the front and rear subcells, respectively. For reference, we fabricated the polymer tandem solar cells that do not benefit from the ICL. This tandem device demonstrated a significantly lower V_OC_ of 0.73 V (data not shown), as well as a notably low J_SC_ compared to the PEDOT:PSS:GO/LZO device. We speculate that this unacceptable performance is because of the formation of an undesired barrier to electron flow in the intended cascade from PC_71_BM to PCDTBT.

Figure 4a and Table 3 demonstrate that the tandem structure improves the performance of the individual subcells (bottom and top cell), since the efficiency of the tandem cells is 30% higher than that of the front subcell and 42% higher than that of the rear subcell. The front subcell generates a photocurrent^28^,^29^ lower than the rear subcell and limits the performance of the tandem cells in series configuration.

The EQE spectra (Fig. 4c) of the constituent single-junction solar cells further confirms current balancing. EQE measurements of the polymer tandem solar cell structures require special precaution due to the coupled light absorption and photocurrent-generation processes in each cell^30^. EQE measurements were taken with two excitation light sources. A 700 nm light optical bias light beam was used to excite only one of the subcells, while a 550 nm light was used to measure the EQE of the other subcell. The EQE spectra demonstrated an excellent balance in photocurrents generated by the front and rear subcells. The EQE spectra closely follow the absorptance spectra of the front and rear subcells, confirming that the photocurrents render from photoactive layers.

In this study, we have fabricated 43 inverted tandem polymer solar cells and measured them using optimized front and bottom subcell thicknesses. Figure S3 demonstrates the histograms of the photovoltaic parameters and it shows that our tandem devices are highly reproducible.

One of the most critical issues for series connected polymer tandem solar cells is current balancing in each subcell. It is accepted that, the rear subcell absorbs the light that is not absorbed by the front subcell and is illuminated under lower light intensities. However, as shown above, the front subcell produces lower photocurrents. This implies that in our tandem, the structure of the extracted photocurrent is almost the same as the photocurrent of the subcell that generates the lowest photocurrent. If the front subcell generates much more photocurrent, the excess electrons cannot recombine with the electrons from the rear subcell and will charge the ICL. This charge will partially compensate for the built-in voltage across the front subcell, until the photocurrent of the front subcell matches the photocurrent of the rear subcell. This results in deteriorated polymer tandem solar cell performance. Thus, to take full advantage of the tandem architecture, the photocurrent generated in the front subcell has to balance the photocurrent of the rear subcell. The photocurrent of the front subcell has to be adjusted until both photocurrents are almost identical.

**Figure 5:**
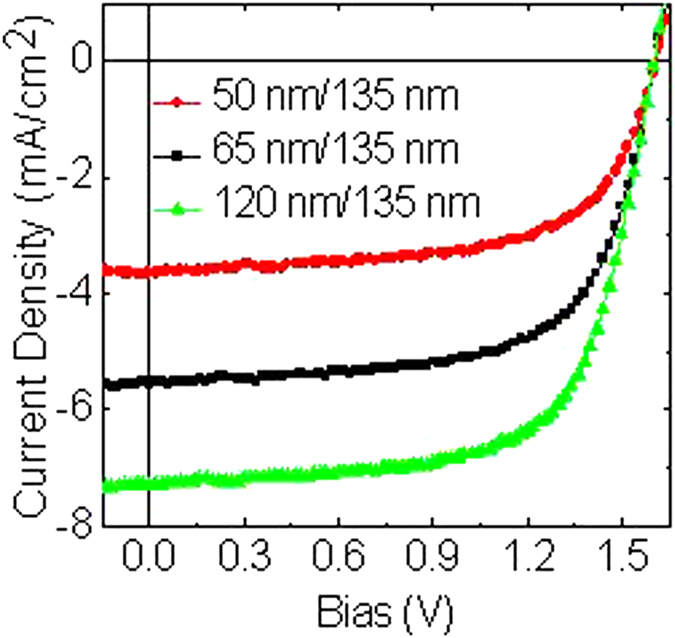
J-V characteristics of inverted tandem cells with different front subcell thicknesses.

Keeping the optimized PBDTT-DPP:PC_71_BM thickness of 135 nm constant, Fig. 5 and Table 4 show the variation of J_SC_ with the PCDTBT:PC_70_BM layer thickness in the proposed tandem device. The thicker the PCDTBT:PC_70_BM active layer, the more light that is transmitted and can be absorbed by the PBDTT-DPP:PC_71_BM layer. Thus it is possible to balance the subcell photocurrent by adjusting the front subcell thicknesses. Clearly, the J_SC_ increases with the increase of the PCDTBT:PC_70_BM thickness. Thicker layers will not only increase the series resistance (R_S_), but also halt carrier transport. Furthermore, according to the working principles of bilayer OPV’s, only the excitons in the PCDTBT:PC_70_BM layer can diffuse the interface for the photocurrent^31^. Therefore, for a very thick PCDTBT:PC_70_BM layer, some excitons will be lost. A very thick PCDTBT:PC_70_BM will also decrease the number of photons reaching the PBDTT-DPP:PC_71_BM layer due to their overlap in the absorption spectra.

**Figure 6:**
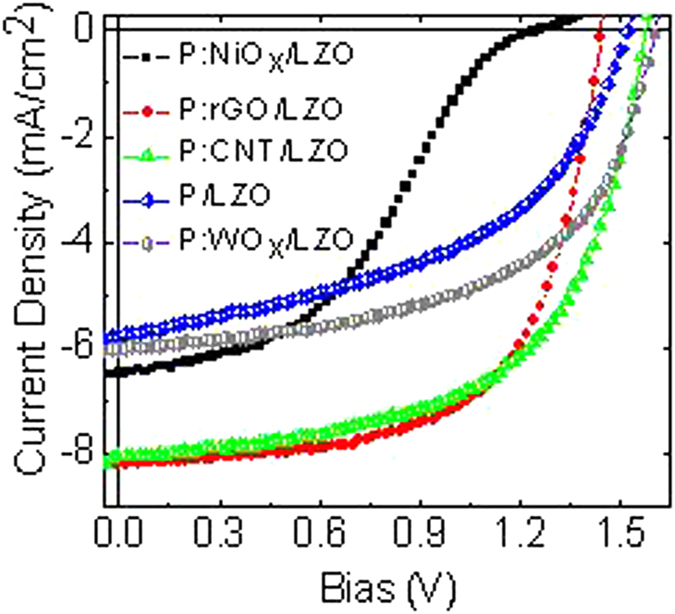
J-V characteristics of inverted tandem polymer solar cells with different ICLs.

The ICL of the polymer tandem solar cells must have Ohmic contact in both the front and rear subcells and induce efficient recombination of charge carriers coming from both cells. In devices made by stacking solution processed films, the ICL must also prevent the solution, during the deposition, of the rear subcell from penetrating into the front subcell^17^. Many appealing and interesting ICL concepts have been put forward lately^32^,^33^,^34^,^35^,^36^,^37^,^38^,^39^,^40^,^41^,^42^. As we know, the ICL not only serves as the charge recombination region for charges coming from the front and rear subcells, but it also ensures the presence of suitable interface energy for efficiently recombining the charges from the subcells. In addition, the ICL must prevent any formation of a reverse built-in potential that will affect the V_OC_ of the polymer tandem solar cells. Hence, the ICL should be optically transparent to avoid any absorption and reflection when projected light passes through the rear subcell back into the front subcell. We expanded our study on polymer tandem solar cells using five different sets of ICLs including i) PEDOT:PSS:NiO_x_/LZO, ii) PEDOT:PSS:rGO/LZO, iii) PEDOT:PSS:CNT/LZO, iv) PEDOT:PSS/LZO, and (v) PEDOT:PSS:WO_x_/LZO. Figure 6 exhibits the J-V characteristics of tandem cells under 100 mW/cm^2^ AM1.5G illumination with different ICLs. The extracted photovoltaic parameters of the respective tandem cells are summarized in Table 5. As shown in Fig. 6, the tandem cells performance varied significantly with different ICLs. This shows that, apart from current balancing between the front and rear subcells, finding a suitable ICL is another crucial issue in designing high performance polymer tandem solar cells. This data demonstrates that polymer tandem solar cells with an ICL of PEDOT:PSS:NiO_x_/LZO and PEDOT:PSS:rGO/LZO have significantly smaller V_OC_’s of 1.24, and 1.44 V which are lower compared to PEDOT:PSS:GO/LZO ICL. We attributed the low V_OC_’s to the energy barrier, which caused a large voltage drop across these ICLs. However, the other three ICLs; PEDOT:PSS:CNT/LZO, PEDOT:PSS:LZO, and PEDOT:PSS:WO_x_/LZO have only slight deviations from the ideal V_OC_ (1.62 V). The PEDOT:PSS:CNT/LZO, and PEDOT:PSS:WO_x_/LZO have the closest V_OC_ values (1.58 and 1.60 V) to the ideal value. In addition, it is worth noting that FF values were bad, indicating that there was a larger internal resistance and inefficient charge extraction and recombination at the ICLs. To ensure the robustness of our ICLs, we tested PEDOT:PSS:GO/LZO ICL with various organic solvents, such as chlorobenzene, chloroform, 1,2-dichlorobenzene, and 1,3,5-trichlorobenzene. Figure S1 shows that our new ICL has a good chemical resistance to prevent any penetration during the deposition of the rear subcell.

However, the anomalous S-shaped curve was observed in the positive bias regime for PEDOT:PSS:NiO_x_/LZO ICL. This indicates that the ICL does not form ohmic contact. In the S-shaped curve, the photocurrent levels turn off with the increase of bias and further increase in the V_OC_ region. This anomalous behavior essentially decreases both the FF and PCE. It has been frequently observed and attributed to (i) the local space charge in the multilayer device^43^, (ii) the defects at the cathode interface and other interfacial behavior^44^,^45^,^46^, and (iii) the strong imbalance of individual charge carrier mobilities^47^. The latest argument is based on vertical phase separation of the two organic components^48^. Villers and co-workers ascribed that the vertical phase segregation was controlled by subtle factors in the solvent evaporation kinetics during the spin-coating process^48^. Gupta and Glatthaar, along with their respective co-workers attributed that the S-shaped curve depends on the quality of the cathode interface and the presence of p-type impurity doping, respectively^49^,^50^.

In order to enable us to evaluate the specific electrical properties of the interfaces, we carried out the impedance spectra measurements in the dark for all fabricated inverted devices with different ICLs. Figure S4 illustrates the Cole-Cole plots of the devices, where the arc of PEDOT:PSS:NiO_x_/LZO is remarkably larger than that of others. The resistance of the ICL reduced in the order of PEDOT:PSS:NiO_x_/LZO, PEDOT:PSS/LZO, PEDOT:PSS:WO_x_/LZO, PEDOT:PSS:CNT/LZO, and PEDOT:PSS:rGO/LZO.

**Figure 7:**
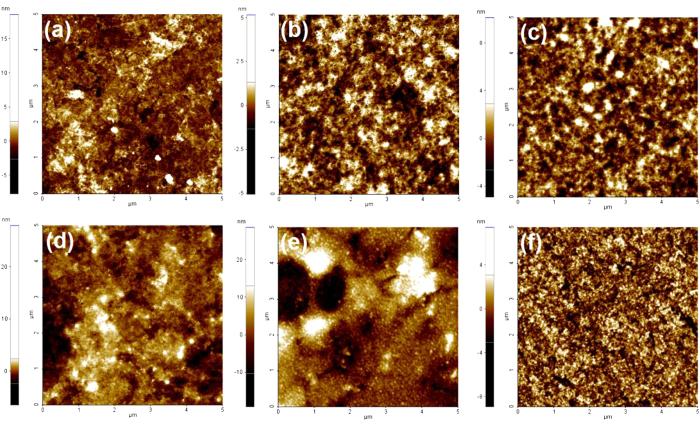
Atomic force images of (a) PEDOT:PSS:NiO_x_/LZO, (b) PEDOT:PSS:rGO/LZO, (c) PEDOT:PSS:CNT/LZO, (d) PEDOT:PSS/LZO, (e) PEDOT:PSS:WO_x_/LZO, and (f) PEDOT:PSS:GO/LZO interconnecting layers deposited onto the front subcells.

To further understand and access in detail, the surface morphologies of the ICL were studied using atomic force microscopy (AFM). All AFM images were taken after the deposition of the corresponding ICLs on the front subcell. As shown in Fig. 7a–e, large aggregations (ca. 150–300 nm) are observed from the film prepared from the PEDOT:PSS:NiO_x_/LZO, PEDOT:PSS:rGO/LZO, PEDOT:PSS:CNT/LZO, PEDOT:PSS/LZO, and PEDOT:PSS:WO_x_/LZO solution. However, no notable aggregation was observed from the film prepared by the PEDOT:PSS:GO/LZO solution (Fig. 7f). The root-mean-square (RMS) roughness value of the PEDOT:PSS:GO/LZO layer was 4.5 nm, which is significantly lower than that of the PEDOT:PSS:NiO_x_/LZO, PEDOT:PSS:rGO/LZO, PEDOT:PSS:CNT/LZO, PEDOT:PSS/LZO, and PEDOT:PSS:WO_x_/LZO layers (67.5, 78.2, 96.4, 88.7, and 87.4 nm). The height profiles of the corresponding film clearly show the difference in the roughness between the films. The height of the PEDOT:PSS:GO/LZO film is around 10 nm and those of the PEDOT:PSS:NiO_x_/LZO, PEDOT:PSS:rGO/LZO, PEDOT:PSS:CNT/LZO, PEDOT:PSS/LZO, and PEDOT:PSS:WO_x_/LZO layers are between of 100–150 nm (not shown).

**Figure 8:**
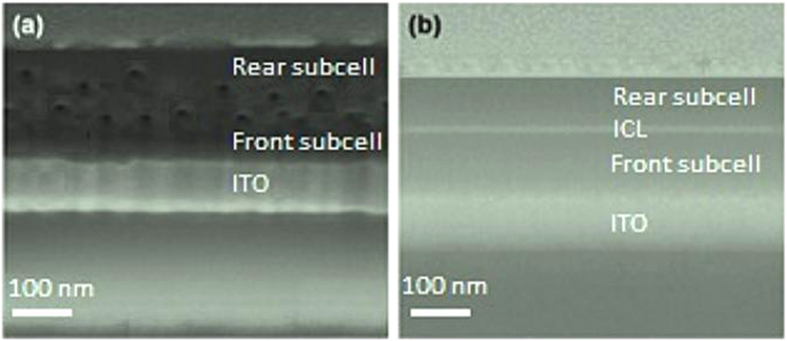
The Focused Ion Beam (FIB) cross-sectional image of the fabricated tandem solar cells incorporating (a) PEDOT:PSS:NiO_x_/LZO, and (b) PEDOT:PSS:GO/LZO interconnecting layers.

To support our AFM images data, the focused ion beam (FIB) images for all polymer tandem solar cells with different ICLs are shown in Fig. 8. We can see that the PEDOT:PSS:GO/LZO (Fig. 8b) obviously separated the front and rear subcells. Unlike PEDOT:PSS:NiO_x_/LZO, interconnecting layers do not have a clear separation of subcells, and large voids (bright region) can be observed (Fig. 8a). Based on the AFM and FIB experiments, it is believed that the dense PEDOT:PSS:GO/LZO ICL helps the separation of subcells and forms better interfacial contact between the PEDOT:PSS:GO and LZO layer. This implies that the recombination efficiency at the PEDOT:PSS:NiO_x_/LZO, PEDOT:PSS:rGO/LZO, PEDOT:PSS:CNT/LZO, PEDOT:PSS/LZO, and PEDOT:PSS:WO_x_/LZO layers will be significantly lower than that at the PEDOT:PSS:GO/LZO ICL. Thus, this leads to a voltage drop across the interface, and results in a lower V_OC_ of the polymer tandem solar cells^51^.

Finally, we concluded our detailed and systematic investigation on polymer tandem solar cells by considering any dependence on aperture. Several authors have previously reported the study of single junction OPVs with and without the presence of aperture or photo-masking^52^. For this purpose, we used an aperture with the same size of our polymer tandem solar cells’ active area (0.04 cm^2^). Figure S2 shows that, in the case without any existence of aperture, the J_SC_ increases about 10% compared to that with aperture (8.53 mA/cm^2^), which leads to a 10.03% jump in PCE. This is probably due to the light piping, where a remarkably huge number of charge carriers outside of the active area also flow toward the electrode. At the same time it influences the photocurrent and the real active area which contributes to the real photocurrent which would be a lot larger than it should be. All summarized photovoltaic parameters are without the existence of aperture, as shown in Table S1. Thus, one effective way to accurately to measure the OPV performance is by introducing the aperture into the active area.

We have demonstrated systematic studies on DPP containing low bandgap polymer PBDTT-DPP in multiple junction OPVs. Combining various different approaches including numerical simulation, morphological, interface, as well as device engineering, our single junction OPV exhibited an improved PCE of nearly 7% for PCDTBT-based OPVs. Moreover, the tandem cells demonstrated a high PCE of 9.02%, which represents the highest small-scale laboratory efficiency. These encouraging observations demonstrate that PBDTT-DPP is a very promising low bandgap polymer for high performance OPVs.

## Experimental Details

### Materials

PEDOT:PSS, PCDTBT, PBDTT-DPP, and PC_70_BM, were purchased from HC Starck, Lumtec, Solarmer, and Sigma Aldrich, respectively and used without further purification. Zinc acetate dihydrate (99.9%) was also purchased from Sigma Aldrich. GO was purchased from Graphene Supermarket.

### LZO solution preparation

LZO films were prepared using the sol-gel method. Zinc acetate dihydrate (Zn(CH_3_COO)_2_.2H_2_O, 99.99%) and Li nitrate (Li(NO_3_), 99%), used as the precursors (molar ratio Li/Zn = (0%–10%), were dissolved in 2-propanol. Ethanolamine (HN(CH_2_CH_2_OH)_2_) was added into the mixed transparent solution as a stabilizer in ethanolamine/zinc acetate molar with a ratio of 1:1.

### PEDOT:PSS:GO solution preparation

The GO and PEDOT:PSS solutions were mixed together with a 1:1 by volume ratio and later stirred for 12 h in a controlled atmosphere.

### Single Junction Fabrication (Front Subcell)

The pre-cleaned ITO substrates were first treated with UV-ozone for 10 min. The LZO solution was spin-coated on the ITO substrates at a spin speed of 1000 rpm for 1 min. The approximate thickness of the film is 30 nm. Then, the active layer was spin coated on the LZO layer. The PCDTBT:PC_70_BM at a 1:4 weight ratio in a 1.25 wt % 1,2 dichlorobenzene solution was then coated on the pre-cleaned ITO-coated glass substrates at 700 rpm for 25 s on top of the LZO layer and baked at 70 °C for 1 h for the first active layer. After spin-coating the active layer, the samples were transferred into the evaporation chamber to fabricate the HTL, and finally the Al electrode. The device area is 0.04 cm^2^. The final device structure consists of ITO/LZO (30 nm)/PCDTBT:PC_70_BM (95 nm)/PEDOT:PSS (10 nm)/Ag (100 nm).

### Single Junction Fabrication (Rear Subcell)

The pre-cleaned ITO substrates were first treated with UV-ozone for 10 min. ETL was spin-coated on the ITO substrates. Then the active layer was spin-coated on the ETL layer. The rear subcell active layer was spin-coated at 4500 rpm. for 1 min from PBDTT-DPP:PC_71_BM (1:2) in 1,2-dichlorobenzene (DCB) (8 mg of PBDTT-DPP/1 mL of solvent) without any processing treatment. After spin-coating the active layer, the samples were transferred into the evaporation chamber to fabricate the (15 nm) MoO_3_/ (100 nm) Ag electrode. The device area is 0.04 cm^2^. The final device consists of ITO/LZO (30 nm)/PBDTT-DPP:PC_71_BM/MoO_3_ (15 nm)/Ag (100 nm).

### Tandem Devices Fabrication

The PCDTBT:PC_70_BM was deposited through a similar process as mentioned above. Then the PEDOT:PSS:GO and LZO were spin-coated on the active layer of the front subcell in sequence. The thickness of PEDOT:PSS:GO and LZO are 40 nm and 10 nm, respectively. Later, the PBDTT-DPP:PC_71_BM was spin-coated on the LZO layer, where the thicknesses of the active layer was controlled by the spin coating speed. Finally, the samples were transferred into the evaporation chamber (1 × 10^−7^ Torr) to fabricate the MoO_3_ (15 nm)/Ag (100 nm) electrode; the device area is 0.04 cm^2^.

### Device Characterization

For tandem solar cells, the layers comprising of LZO/PCDTBT:PC_70_BM/PEDOT:PSS:GO/LZO/PBDTT-DPP:PC_71_BM were electrically isolated using toluene and methanol along the perimeter, as defined by the area of the top electrode. This isolation prevents fringing effects and also eliminates over estimation of the photocurrents generated by the tandem cell. During the measurements and stability tests, a shadow mask (0.04 cm^2^) with a single aperture was placed onto the tandem solar cells in order to identify its photoactive area. The current density–voltage (*J*–*V*) characteristics were recorded with a Keithley 2410 source unit. The EQE measurements were performed using an EQE system (Model 74000) obtained from Newport Oriel Instruments USA and HAMAMATSU calibrated silicon cell photodiodes were used as the reference diode. The wavelength was controlled with a monochromator 200–1600 nm.

## Additional Information

**How to cite this article**: da Silva, W. J. *et al.* High performance polymer tandem solar cell. *Sci. Rep.* **5**, 18090; doi: 10.1038/srep18090 (2015).

## Supplementary Material

Supplementary Information

## Figures and Tables

**Table 1:** Device performance of PCDTBT:PC_70_BM-based inverted single junction solar cells with different BHJ layer thicknesses and different HTLs.

Active layer thickness (nm)	HTL	J_sc, exp_ (mA/cm^2^)	J_sc, sim_ (mA/cm^2^)	V_oc_(V)	FF (%)	PCE (%)
95	PEDOT:PSS:GO	11.13	11.20	0.88	70.78	6.93
105	PEDOT:PSS:GO	10.98	11.02	0.88	70.67	6.83
120	PEDOT:PSS:GO	10.18	10.21	0.87	69.97	6.27
95	PFN	5.31	5.32	0.87	55.35	2.52
95	Ca	9.78	9.80	0.87	64.04	4.52

**Table 2:** Device performance of PBDTT-DPP:PC_71_BM-based inverted single junction solar cells with different BHJ layer thicknesses and different ETLs.

Active layer thickness (nm)	ETL	J_sc_ (mA/cm^2^)	J_sc, sim_ (mA/cm^2^)	V_oc_ (V)	FF (%)	PCE (%)
135	LZO	13.08	13.23	0.74	65.69	6.36
145	LZO	12.45	13.94	0.72	68.11	6.24
155	LZO	11.93	13.43	0.73	63.50	5.71
135	ZnO:TiO_x_	12.70	13.67	0.74	58.90	5.67
135	AZO	11.00	12.21	0.73	55.80	4.70

**Table 3:** Device performance of front, rear, and inverted tandem cells.

Structure	J_sc_ (mA/cm^2^)	V_oc_(V)	FF (%)	PCE (%)
Front	11.13	0.88	70.78	6.93
Rear	13.08	0.74	65.69	6.36
Tandem	8.53	1.60	66.14	9.02

**Table 4:** Device performance of inverted tandem polymer solar cells with different front subcell thicknesses.

Active layer thickness (Front/Rear) [nm]	J_sc_ (mA/cm^2^)	V_oc_(V)	FF(%)	PCE(%)
50/135	3.65	1.60	63.01	3.68
65/135	5.55	1.60	65.58	5.82
120/135	7.30	1.60	66.00	7.72

**Table 5:** Device performance of inverted tandem polymer cells with different ICLs.

Interconnecting layer	J_sc_(mA/cm^2^)	V_oc_(V)	FF (%)	PCE (%)
PEDOT:PSS:NiO_x_/LZO	6.48	1.24	40.44	3.25
PEDOT:PSS:rGO/LZO	8.20	1.44	62.88	7.43
PEDOT:PSS:CNT/LZO	8.05	1.58	58.22	7.41
PEDOT:PSS/LZO	5.78	1.52	47.70	4.19
PEDOT:PSS:WO_x_/LZO	6.03	1.60	55.37	5.34
