# Supplementary material for: High performance polymer tandem solar cell
Source: Sci Rep. 2015 Dec 16;5:18090. doi: 10.1038/srep18090 (PMC4680961; doi:10.1038/srep18090)
Supplement: Supplementary Information [file srep18090-s1.doc]

Supporting Information

**High performance polymer tandem solar cell**

Wilson Jose da Silva,* Fabio Kurt Schneider, Abd. Rashid bin Mohd Yusoff, Jin Jang*

[*] Prof. Dr. A. B. R. M. Yusoff, Corresponding-Author, Prof. Dr. J. Jang
Department of Information Display, Advanced Display Research Center, Kyung Hee University, Dongdaemoon-gu, 130-701 Seoul, South Korea
E-mail: jjang@khu.ac.kr

Dr. W. J. da Silva, Dr. F. K. Shneider
Universidade Tecnologica Federal do Parana, GPGEI – Av. Sete de Setembro, 3165 – CEP 80230-901, Curitiba, Parana, Brazil

Email:wjsilva2000@yahoo.com.br


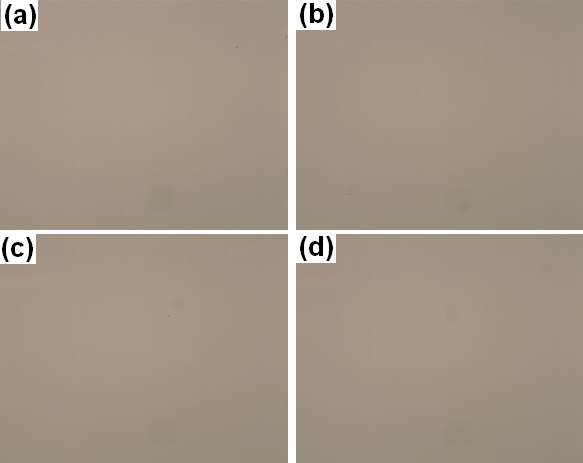


**Figure S1.** PEDOT:PSS:GO/LZO interconnecting layer treated with various different solvents, however, no damage can be seen. (a) Chlorobenzene. (b) Chloroform. (c) 1,2-dichlorobenzene. (d) 1,3,5-trichlorobenzene.

**
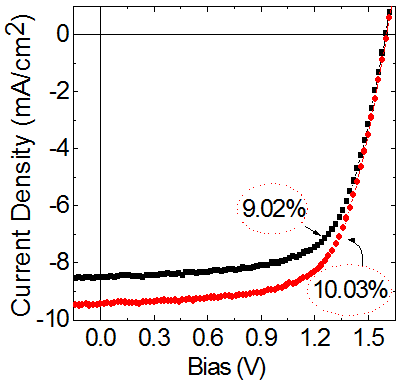
**

**Figure S2.** J-V characteristics of inverted tandem polymer solar cells with and without the existence of aperture.


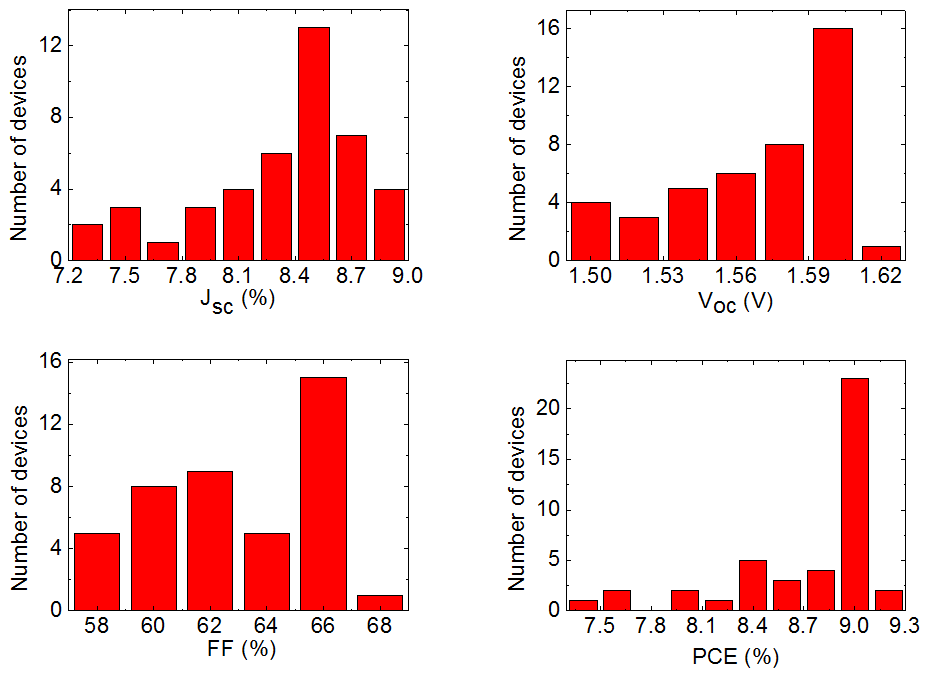


**Figure S3.** Histogram of inverted tandem polymer solar cells parameters measured for 43 separate devices under 1 sun, AM 1.5G illumination (25 °C, 100 mW/cm^2^). J_SC_ (a), V_OC_ (b), FF (c), and PCE (d).


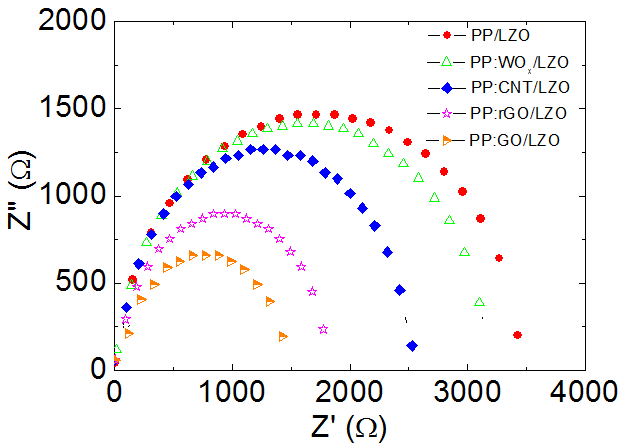


**Figure S4.** Impedance spectra of inverted tandem polymer solar cells with different interconnecting layers in the dark.

**Table S1. Device performance of inverted tandem polymer solar cells with and without the existence of aperture.**

| Structure | J_sc_  (mA/cm^2^) | V_oc_  (V) | FF  (%) | PCE  (%) |
| --- | --- | --- | --- | --- |
| Without aperture | 9.44 | 1.60 | 66.42 | 10.03 |
| With aperture | 8.53 | 1.60 | 66.14 | 9.02 |
